# Transcriptional repressor Gal80 recruits corepressor complex Cyc8–Tup1 to structural genes of the *Saccharomyces cerevisiae GAL* regulon

**DOI:** 10.1007/s00294-021-01215-x

**Published:** 2021-10-07

**Authors:** Julia Lettow, Rasha Aref, Hans-Joachim Schüller

**Affiliations:** 1 Center for Functional Genomics of Microbes, Abteilung Molekulare Genetik und Infektionsbiologie, Felix-Hausdorff-Str. 8, 17487 Greifswald, Germany; 2 Department of Genetics, Faculty of Agriculture, Ain Shams University, Shoubra El-Khaymah, Cairo, 11241 Egypt

**Keywords:** *Saccharomyces cerevisiae*, *GAL* regulon, Gal80, Corepressor, Cyc8, Tup1

## Abstract

Under non-inducing conditions (absence of galactose), yeast structural genes of the *GAL* regulon are repressed by Gal80, preventing interaction of Gal4 bound to UAS_GAL_ promoter motifs with general factors of the transcriptional machinery. In this work, we show that Gal80 is also able to interact with histone deacetylase-recruiting corepressor proteins Cyc8 and Tup1, indicating an additional mechanism of gene repression. This is supported by our demonstration that a lexA–Gal80 fusion efficiently mediates repression of a reporter gene with an upstream lexA operator sequence. Corepressor interaction and in vivo gene repression could be mapped to a Gal80 minimal domain of 65 amino acids (aa 81-145). Site-directed mutagenesis of selected residues within this domain showed that a cluster of aromatic-hydrophobic amino acids (YLFV, aa 118-121) is important, although not solely responsible, for gene repression. Using chromatin immunoprecipitation, Cyc8 and Tup1 were shown to be present at the *GAL1* promoter in a wild-type strain but not in a *gal80* mutant strain under non-inducing (derepressing) growth conditions. Expression of a *GAL1–lacZ* fusion was elevated in a *tup1* mutant (but not in a *cyc8* mutant) grown in derepressing medium, indicating that Tup1 may be mainly responsible for this second mechanism of Gal80-dependent gene repression.

**Supplementary Information:**

The online version contains supplementary material available at 10.1007/s00294-021-01215-x.

## Introduction

Transcriptional repression in eukaryotes can be executed by several molecular mechanisms such as prevention of nuclear import of an activator, inhibition of its DNA binding, or deactivation of transcriptional activation domains. However, generating a chromatin structure being refractory against access of transcription factors is of general importance for eukaryotic gene repression (Courey and Jia 2001). This is achieved by gene-specific repressor proteins utilizing a limited number of corepressors which finally recruit chromatin modifying enzymes such as histone deacetylases (HDACs), leading to a less-accessible structure of chromatin. Indeed, Rpd3 as the major HDAC in *S. cerevisiae* was initially described as a negative regulator of potassium uptake (“reduced potassium dependency”; Taunton et al. 1996; Vidal et al. 1991). Two corepressor complexes in yeast (Sin3/Rpd3L and Cyc8–Tup1) are required as scaffolds to bring HDACs in close contact to gene-specific repressor proteins (Adams et al. 2018; Malavé and Dent 2006).

Mutations affecting Cyc8 (= Ssn6) and Tup1 have been initially described as pleiotropically defective for diverse cellular functions such as glucose repression (Treitel and Carlson 1995), hypoxia (Mennella et al. 2003), control of mating type (Keleher et al. 1992), and DNA damage repair (Huang et al. 1998). Neither Cyc8 nor Tup1 is able to specifically bind DNA, but depends on interaction with sequence-specific DNA-binding proteins (Mig1, Rox1, α2, and Crt1, among others) to trigger gene repression. Structural repetitions within Cyc8 (10 tetratricopeptide repeats, TPR, found at the N-terminus; Tzamarias and Struhl 1995) and Tup1 (7 WD40 repeats at the C-terminus; Zhang et al. 2002) are indispensable for repression function of the complex with a stoichiometry of one Cyc8 subunit associated with four Tup1 subunits (Varanasi et al. 1996). The corepressor complex Cyc8–Tup1 may inhibit gene activation by contacting multiple HDACs (Rpd3, Hda1, Hos1, and Hos2; Davie et al. 2003; Watson et al. 2000; Wu et al. 2001), leading to a more compact chromatin which is less accessible for the transcriptional machinery, and by masking activation domains, thereby preventing recruitment of coactivators such as SWI/SNF, RSC, SAGA, and mediator (Wong and Struhl 2011). However, comparative expression studies of wild-type and *cyc8*/*tup1* mutants as well as interaction experiments with activator proteins indicate a more complex function for Cyc8–Tup1 which may also exhibit a positive function for gene control (Zhang and Guarente 1994; Papamichos-Chronakis et al. 2002; Kliewe et al. 2017).

The *GAL* regulon is a paradigm for differential gene expression in eukaryotes and for interactions of gene-specific regulators with pleiotropic factors (reviewed by Lohr et al. 1995; Traven et al. 2006). Depending on the quality of the carbon source available, three regulatory situations can be distinguished. (1) Glucose repression: In the presence of glucose as a favorable substrate, binding sites of the zinc-finger repressor Mig1 upstream of activator gene *GAL4* and *GAL* structural genes strongly contribute to glucose repression of the *GAL* regulon (Nehlin et al. 1991; Johnston et al. 1994) which is triggered by Mig1-dependent recruitment of Cyc8–Tup1 (Treitel and Carlson 1995). (2) Non-inducing conditions (derepression): Deactivation of Mig1 by Snf1 increases biosynthesis of activator Gal4 which binds to UAS_GAL_ motifs, but is still inhibited as a result of masking its C-terminal activation domain by repressor Gal80 (Johnston et al. 1987; Ma and Ptashne 1987), preventing the recruitment of basal transcription factors and histone acetyltransferase complexes (TBP, TFIIB, SAGA, NuA4; Wu et al. 1996; Carrozza et al. 2002). (3) Galactose induction: Although mechanistic details are still controversial, Gal3 as the galactose sensor binds to Gal80, leading to a conformational shift or even its dissociation from Gal4 which makes its activation domain accessible for various coactivators (Leuther and Johnston 1992; Melcher and Johnston 1995; Yano and Fukasawa 1997; Jiang et al. 2009).

By emphasizing the hindrance of the Gal4 activation domain by Gal80, the previous work focused on its function as an anti-activator. In a systematic screen for repressor–corepressor interactions, we could show that Gal80 is also able to recruit Cyc8–Tup1. We demonstrate that an internal Gal80 domain of 65 amino acids can bind to both Cyc8 and Tup1. The same domain mediates strong transcriptional repression in a reporter system which is independent of Gal4. We thus provide evidence for an additional mechanism preventing activation of *GAL* genes under non-inducing conditions.

## Materials and methods

### Yeast strains, media, and growth conditions

*S. cerevisiae* strain RTS-lexA (*MAT*α *leu2 trp1 his3 ura3::lexA*_*Op*_*-CYC1-lacZ::URA3*) was derived from regulatory wild-type JS91.15-23 (Jäschke et al. 2011) by transformation with an integrating variant of plasmid pJK1621 (Keleher et al. 1992; contains 4 lexA operator sites upstream of the native *CYC1* promoter). Strains JS167 (wild-type), JS05.2-8 (*cyc8*∆), and JS95.7-1 (*tup1*∆) used for comparative expression studies of a *GAL1–lacZ* reporter gene are isogenic to JS91.15-23. Null mutant alleles were introduced into wild-type strains using disruption plasmids pJL78 (*gal80*∆*::LEU2*; this work), pJN41(*mig1*∆*::URA3*; Nehlin and Ronne 1990), pDSB (*cyc8*∆*::LEU2*; Trumbly 1988), and pFW36 (*tup1*∆*::TRP1*; Williams and Trumbly 1990). Complete genotypes of all strains used in this work are shown in Supporting Online Table S1.

Strains were cultivated in synthetic complete media with 2% glucose (repressing conditions), 0.1% glucose + 1% lactate (derepressing/non-inducing conditions), or 2% galactose (inducing conditions). Supplementation with 0.1% glucose (which is completely consumed before cell harvest) supports growth under derepressing conditions.

### Plasmid constructions and site-directed mutagenesis

GST and HA_3_fusions were synthesized in *E. coli*, using expression plasmids pGEX-2TK (tac promoter-operator; GE Healthcare) and pASK-IBA5-HA3 (tet promoter-operator; IBA, Göttingen, Germany; modified by insertion of HA-encoding sequences), respectively. Length variants of *GAL80* were amplified by PCR using specific primers (cf. Supporting Online Table S1) and fused behind GST. To obtain epitope-tagged corepressors Cyc8 and Tup1 in bacterial protein extracts, plasmids pFK77 (HA_3_-*CYC8*; encoding aa 1-398 representing the TPR-containing domain) and pFK76 (HA_3_-*TUP1*; full length) were used. HA_3_-tagged Sin3 (full-length) was synthesized in yeast, using expression plasmid pCW117 (Wagner et al. 2001).

Missense mutations were introduced into the *GAL80* coding sequence at specific positions, using the QuikChange site-directed mutagenesis kit (Agilent/Stratagene) and pairs of mutagenic primers, allowing replacement of natural codons against an alanine-specific codon (primer sequences shown in Supporting Online Table S1). The authenticity of *GAL80* length and mutational variants was verified by DNA sequencing (LGC Genomics, Berlin, Germany).

To assay for gene repression in vivo, plasmid RTS-lexA was used (constructed and kindly provided by F. Kliewe). This plasmid encodes the HA_3_-tagged DNA-binding domain of bacterial repressor lexA, followed by the nuclear localization sequence of T antigen from SV40 (MPKKKRLV) and a versatile cloning site which was used for insertion of *GAL80* length and mutational variants.

Plasmid names and genetic markers are compiled in Supporting Online Table S1.

### In vitro* interaction assays (GST pull-down)*

To synthesize GST- and HA-tagged proteins in *E. coli*, strain BL21 (Stratagene/ Agilent) was used. Gene promoters *tac* and *tet* were induced with 1 mM IPTG and 0.2 mg/l anhydrotetracycline, respectively. For maximal expression of *MET25*-activated gene fusions in *S. cerevisiae*, yeast transformants were cultivated in the absence of methionine.

GST fusion proteins were released from induced *E. coli* cells by sonication and similar amounts were bound to glutathione (GSH) sepharose (according to GST enzyme assays in crude extracts). GST fusions were incubated with yeast or bacterial total protein extracts containing HA fusions of Cyc8, Tup1 or Sin3. Following intensive washing reactions under conditions of intermediary stringency (Wagner et al. 2001), sepharose-bound GST fusions were released by adding free GSH (10 mM). Eluted proteins were separated by SDS-PAGE and transferred to a PVDF membrane which was finally treated with anti-HA–peroxidase conjugate, allowing detection of HA fusion proteins with POD chemiluminescent substrate (antibody conjugate and substrate from Sigma-Aldrich).

### Chromatin immunoprecipitation (ChIP)

Chromatin immunoprecipitation analyses were essentially performed as previously described (Kliewe et al. 2017). Using plasmid pU6H3HA (contains a His_6_-HA_3_-*kanMX* cassette; De Antoni and Gallwitz 2000) and gene-specific primers, gene replacement cassettes were amplified to modify authentic chromosomal loci *CYC8* and *TUP1*, such that they express His-tagged Cyc8 and Tup1, respectively. The resulting strains FKY12 (*CYC8*-His_6_-HA_3_) and JuLY1 (*TUP1*-His_6_-HA_3_) were derived from the proteinase-deficient strain C13-ABY.S86 by transformation with gene-specific replacement cassettes, selecting for resistance against geneticin. These strains together with isogenic *gal80*∆, *mig1*∆, and *gal80*∆ *mig1*∆ derivatives were grown in synthetic medium and 1% lactate as a non-inducing carbon source until mid-log phase. Following cross-linking by treatment with formaldehyde and quenching of the reaction by addition of glycine, cells were lysed and sonicated 5 times for 30 s to shear chromatin (Bandelin Sonoplus UW 70 microtip, 35% power). The lysate was cleared by centrifugation and incubated for at least 30 min with His-Tag Dynabeads® (Invitrogen/Dynal®). Unbound proteins were removed by intensive washing. Affinity-purified proteins together with cross-linked DNA were then eluted with a buffer-containing 300 mM imidazole and cross-linking was reversed by heat treatment (65 °C overnight). After degradation of proteins with proteinase K, DNA was precipitated, purified, and subsequently analyzed by end-point-PCR (38 amplification cycles) and real-time PCR, using specific primers against *GAL1* promoter (Gal1-F170 and Gal1-R450; -450/-170) or *ACT1* gene (Act1-FOR3 and Act1-REV3; + 841/ + 1165) as a control. For analysis of input lysates, 5% of immunoprecipitates were used. Quantitative PCR experiments were performed in triplicate, using the Applied Biosystems StepOnePlus Real-Time PCR system (AB Germany, Darmstadt) together with the SYBR® Green-containing PCR master mix Luna MM (New England BioLabs). Absolute copy numbers of fragments were calculated from Ct values using standard curves according to the recommendations of the manufacturer.

## Results

### Gal80 repressor physically interacts with corepressors Cyc8 and Tup1

**Fig. 1:**
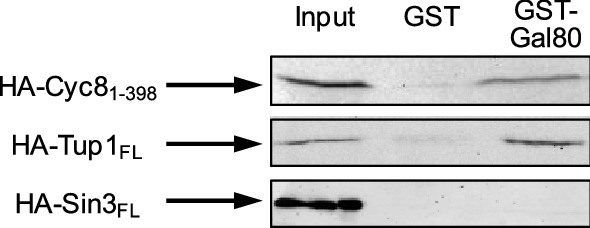
In vitro assays for interaction of Gal80 with pleiotropic corepressors Cyc8, Tup1, and Sin3. Full-length fusion protein GST-Gal80 (encoded by pRAR41) was synthesized in *E. coli*, immobilized on GSH sepharose, and incubated with protein extracts containing epitope-tagged Cyc8 (residues 1-398 comprising TPR motifs 1-10, encoded by pFK77), Tup1 (FL, full-length; encoded by pFK76), or Sin3 (FL, full-length; encoded by pCW117). Interaction assays with GST (pGEX-2TK) served as negative controls (middle lanes)

We have previously shown that gene-specific repressor proteins may execute their function by physically contacting several corepressor complexes (Jäschke et al. 2011; Aref and Schüller 2020). Gal80 is usually considered as a repressor preventing activation of UAS_GAL_-dependent structural genes under non-inducing conditions by masking of the Gal4 activation domain (Johnston et al. 1987; Ma and Ptashne 1987). In a systematic screen for repressor–corepressor interactions, we constructed a GST fusion of repressor Gal80 and used it for in vitro-binding assays with epitope-tagged pleiotropic corepressors Sin3, Cyc8, and Tup1. GST-Gal80 (full-length) was immobilized using glutathione (GSH) sepharose and incubated with protein extracts containing corepressor proteins. As shown in Fig. 1, Gal80 was able to interact with Cyc8 and Tup1 but not with Sin3. Since both corepressors were synthesized in *E. coli*, we also conclude that Gal80 can directly interact with Cyc8 and Tup1 without contribution of additional yeast proteins.

**Fig. 2:**
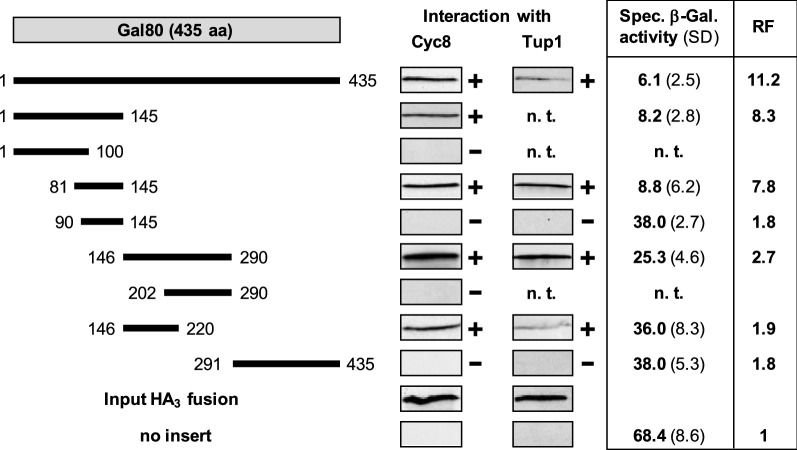
Mapping of Gal80 domains mediating in vitro interaction with corepressors Cyc8 and Tup1 and gene repression in vivo. Gal80 length variants were fused with GST, immobilized on GSH sepharose and incubated with bacterial protein extracts, containing HA-Cyc8 (pFK77) or HA-Tup1 (full-length, pFK76). The following GST-Gal80 expression plasmids were used: pRAR41 (aa 1-435), pRAR53 (aa 1-145), pRAR68 (aa 1-100), pJL13 (aa 81-145), pJL29 (aa 90-145), pJL48 (aa 146-290), pRAR70 (aa 202-290), pJL14 (aa 146-220), and pJL52 (aa 291-435). To measure gene repression in vivo, strain RTS-lexA (reporter gene lexA_Op_-*CYC1-lacZ*) was individually transformed with plasmids encoding the following lexA–Gal80 fusions: pJL17 (aa 1-435), pJL35 (aa 1-145), pJL21 (aa 81-145), pJL25 (aa 90-145), pJL36 (aa 146-290), pJL22 (aa 146-220), and pJL40 (aa 291-435). Empty vector pRT-lexA lacking effector domains served as a negative control for maximal reporter gene expression. Specific β-galactosidase activities are given in nmol oNPG hydrolyzed per min per mg of protein. n. t., not tested; RF, repression factor; + , in vitro interaction; -, no interaction

This result indicates that covering of the transcriptional activation domain of Gal4 under non-inducing growth conditions may be not the sole function of Gal80. In addition, its interaction with the Cyc8–Tup1 complex could subsequently allow recruitment of histone deacetylases such as Rpd3, Hda1, Hos1, and Hos2 to chromosomal sites where Gal4–Gal80 bind, leading to a less-accessible structure of local chromatin. We thus investigated whether fusion of Gal80 to the DNA-binding domain of lexA influences gene expression of a lexA_Op_-containing reporter gene (lexA_Op_-*CYC1-lacZ*) which had been integrated into yeast chromosomal DNA to give strain RTS-lexA. Indeed, transformants of RTS-lexA containing a lexA–Gal80 fusion could efficiently repress this reporter gene by a factor of 11.2 (plasmid pRT-lexA lacking Gal80 sequences as a reference; Fig. 2). Since this reporter system is completely devoid of Gal4 domains, Gal80 must be able to negatively influence gene expression by an independent mechanism.

### Internal domain of Gal80 binds to corepressors and mediates gene repression

For a more precise analysis, we next investigated whether Gal80 binding to Cyc8 and Tup1, respectively, and gene repression can be assigned to a defined region. Previous work has shown that Gal80 is involved in several protein–protein interactions (dimerization, binding to Gal3 and Gal4) and also contains an NAD(P) dinucleotide (Kumar et al. 2008; Lavy et al. 2012). We thus constructed GST-Gal80 length variants and used them for interaction studies with HA-Cyc8 and HA-Tup1, respectively. As shown in Fig. 2, in vitro experiments provided evidence for two non-overlapping internal regions of Gal80 which were able to bind to both corepressor proteins (aa 81-145 and aa 146-220 of Gal80). The C-terminus of Gal80 which is indispensable for dimerization and binding to Gal3 and Gal4 could not interact with Cyc8 and Tup1. To verify these results by in vivo studies, selected *GAL80* truncations were fused with lexA and transformed into reporter strain RTS-lexA. Importantly, Gal80 length variant aa 81-145 could not only interact with Cyc8 and Tup1 but was also able to mediate gene repression almost as efficiently as full-length Gal80 (repression factor 7.8). We thus consider aa 81-145 of Gal80 as a repression-mediating domain. Although length variants aa 146-290 and aa 146-220 could also bind to Cyc8 and Tup1 in vitro, gene repression in vivo was clearly less effective (RF 2.7 and 1.9). Possibly, the position of Gal80 used for construction of these lexA fusion proteins may cause folding problems, preventing the formation of an autonomous domain which should be able to recruit corepressors in vivo as a prerequisite for efficient gene repression.

### A cluster of hydrophobic amino acids is important for corepressor binding and gene repression

Previously, we performed a mutational analysis of the domain of repressor Opi1 responsible for interaction with corepressors Sin3 and Cyc8 and could show that certain hydrophobic amino acids are indispensable for in vitro interaction and in vivo gene repression (Jäschke et al. 2011). Interestingly, the internal domain aa 81-145 of Gal80 shown to bind to Cyc8 and Tup1 and mediating gene repression also contains a short sequence with a hydrophobic–amphipathic pattern (LKYLFVEWALACSL; aa 116-129). We thus replaced selected amino acids within this sequence with alanine and subsequently investigated the influence of the variants obtained on corepressor interaction and gene repression in vivo.

**Fig. 3:**
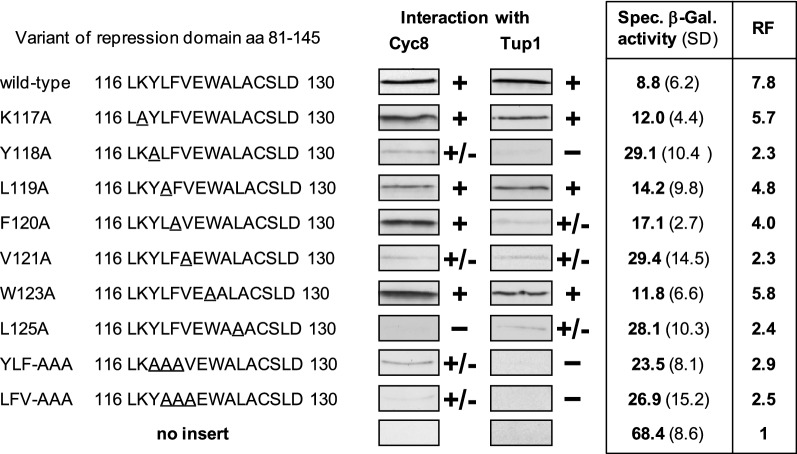
Mutational analysis of selected amino acids within the Gal80 repression-mediating domain. Gal80 variants comprising aa 81-145 were fused with GST, immobilized on GSH sepharose, and incubated with bacterial protein extracts, containing HA-Cyc8 (pFK77) or HA-Tup1 (full-length, pFK76). The following GST-Gal80 expression plasmids were used: pJL13 (aa 81-145, wild-type), pJL57 (K117A), pJL58 (Y118A), pJL59 (L119A), pJL55 (F120A), pJL60 (V121A), pJL56 (W123A), pJL61 (L125A), pJL62 (YLF-AAA, pos. 118-120), and pJL63 (LFV-AAA, pos. 119-121). To measure gene repression in vivo, strain RTS-lexA (reporter gene lexA_Op_-*CYC1-lacZ*) was individually transformed with plasmids encoding the following lexA–Gal80 fusions: pJL21 (aa 81-145, wild-type), pJL42 (K117A), pJL43 (Y118A), pJL44 (L119A), pJL37 (F120A), pJL45 (V121A), pJL38 (W123A), pJL47 (L125A), pJL53 (YLF-AAA, pos. 118-120), and pJL65 (LFV-AAA, pos. 119-121). Empty vector pRT-lexA lacking effector domains served as a negative control for maximal reporter gene expression. Specific β-galactosidase activities are given in nmol oNPG hydrolyzed per min per mg of protein. n. t., not tested; RF, repression factor; + , in vitro interaction; ± , weakened but residual interaction; -, no interaction

As is apparent from Fig. 3, single-point mutations Y118A, L119A, F120A, V121A, and L125A weaken interaction with Cyc8 and/or Tup1 and lead to significantly reduced gene repression. In contrast, no pronounced alteration was observed after exchange of the basic residue K117. The same is true for W123 which has been identified as a residue involved in NAD(P) binding to Gal80 (Kumar et al. 2008). Since no single mutation completely abolished interaction with corepressors Cyc8 and Tup1, some functional redundancy among hydrophobic amino acids may occur. We thus constructed triple mutations of consecutive residues, giving Gal80 variants YLF-AAA and LFV-AAA (positions 118-120 and 119-121, respectively). In vitro binding of both mutated Gal80 repression domains to Cyc8 and Tup1 and gene repression in vivo were also weaker than observed with the wild type. However, the functional loss of the triple mutants was not significantly more apparent than observed with the single mutants. It can be concluded that the cluster YLFV of aromatic-hydrophobic amino acids (aa 118-121) is important but not solely responsible for the functioning of Gal80-dependent gene repression mediated by Cyc8–Tup1.

### Gal80-dependent corepressor recruitment to UAS_GAL_ under non-inducing growth conditions

Our findings provide evidence that Gal80 under non-inducing conditions (absence of glucose and galactose, and presence of an alternative carbon source such as lactate or ethanol) not only masks the Gal4 activation domain but also recruits the corepressor complex Cyc8 + Tup1. Chromatin immunoprecipitation (ChIP) was thus used to investigate whether Cyc8 and Tup1 are indeed detectable at the UAS_GAL_-containing *GAL1* promoter under these conditions. Although Mig1-dependent binding of Cyc8–Tup1 to glucose-regulated promoters (such as *GAL1*) has been described as a mechanism of gene repression (Treitel and Carlson 1995), Mig1 must not be the sole regulator responsible for corepressor recruitment (Papamichos-Chronakis et al. 2004). We constructed strains encoding epitope-tagged variants of *CYC8* and *TUP1* at their native genomic position and subsequently introduced *gal80* and *mig1* null mutations individually and both combined. Zinc finger repressors Mig2 and Mig3 were not considered for our analysis, because these proteins do not influence *GAL1* expression (Westholm et al. 2008); in addition, no interaction with Cyc8–Tup1 has been reported for these proteins.

**Fig. 4:**
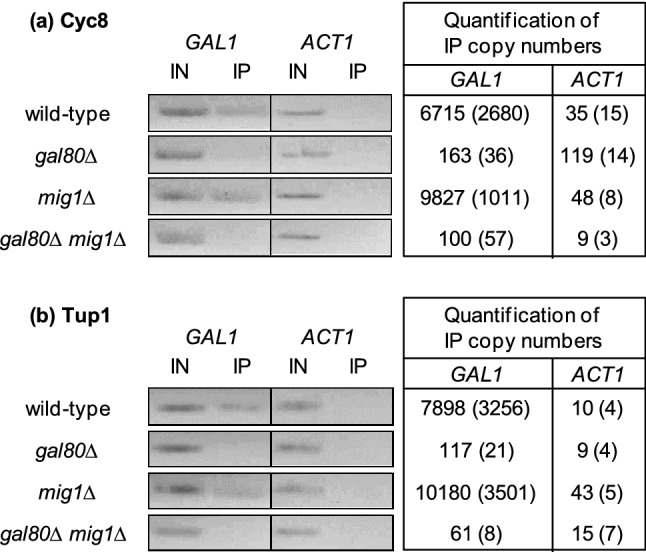
Chromatin immunoprecipitation analysis (ChIP) of *GAL1* promoter fragments from cells grown under non-inducing conditions. **a** Strains FKY12 (wild-type), JuLY2 (*gal80*∆), JuLY7 (*mig1*∆), and JuLY4 (*gal80*∆ *mig1*∆) each encoding a His-tagged variant of *CYC8* were cultivated in SCLac (0.1% glucose + 1% lactate) and used for ChIP analysis. **b** Strains JuLY1 (wild-type), JuLY3 (*gal80*∆), JuLY8 (*mig1*∆), and JuLY5 (*gal80*∆ *mig1*∆) each encoding a His-tagged variant of *TUP1* were cultivated in SCLac (0.1% glucose + 1% lactate) and used for ChIP analysis. Chromatin fragments obtained by ultrasonic treatment were incubated with His-Tag Dynabeads® to concentrate His-tagged proteins. Promoter fragments for *GAL1* and *ACT1* (negative control) were qualitatively analyzed by end-point PCR and quantified by real-time PCR (copy numbers of fragments in immunoprecipitates) using specific primers. *IN* input control of total chromatin fragments, *IP* immunoprecipitate (samples obtained by affinity purification)

In ChIP analyses with cells of wild-type strains cultivated under non-inducing conditions (1% lactate), Cyc8 and Tup1 could indeed be detected at the *GAL1* promoter (Fig. 4; depicting ChIP experiments by qualitative end-point analysis and quantification by real-time PCR). Similar signal intensities were obtained with *mig1* single-deletion strains, demonstrating that Mig1 is not involved in corepressor recruitment under these conditions. In contrast, with isogenic strains devoid of Gal80, copy numbers of the *GAL1* promoter were reduced 41-fold (epitope-tagged Cyc8) and 67-fold (epitope-tagged Tup1), supporting the view that Gal80 is responsible for recruiting Cyc8 and Tup1 to *GAL* gene promoters under non-inducing conditions. In the absence of Gal80 and Mig1, even less *GAL1* promoter sequences could be detected (67-fold and 130-fold reduced concentrations with labeled Cyc8 and Tup1, respectively). Since the role of Mig1 for recruitment of Cyc8–Tup1 has been questioned by Papamichos-Chronakis et al. (2004), our results show that instead Gal80 should be responsible for this function, at least under non-inducing conditions.

### Negative and positive influence of Cyc8–Tup1 on regulation of *GAL* genes

We finally studied the influence of null mutations *cyc8*∆ and *tup1*∆ on the regulated expression of a *GAL1–lacZ* reporter gene. For comparison, expression of a *TPI1–lacZ* fusion was investigated in parallel. *TPI1* encodes a glycolytic enzyme, expression of which is considered as only slightly affected by variation of physiological carbon sources (Moore et al. 1991). As shown in Table 1, the expected derepression (17-fold) and induction (about 400-fold) of *GAL1* was measured in the wild-type strain. Importantly, individual loss of Cyc8 and Tup1 did not influence *GAL1* in the same way: While reporter gene expression in the *cyc8*∆ strain was below the wild-type level under all conditions tested, the *tup1*∆ strain showed elevated activity under repressing and derepressing (but not under inducing) conditions. These results support previous findings on the dual role of Cyc8–Tup1 for the expression of *GAL* genes (Papamichos-Chronakis et al. 2002) and argue for a mainly positive function of Cyc8. In contrast, Tup1 must be considered as a negative factor under repressing and derepressing conditions, while full galactose induction also requires Tup1 (positive factor). As a result, the ratio of *GAL1* expression under inducing versus repressing conditions was significantly lowered from 400 (wild-type) to 80 (*tup1*∆).

**Table 1:** Influence of Cyc8 and Tup1 on expression of *GAL1* with different supply of carbon sources

Strain	Genotype	pKH29 (*GAL1–lacZ*)			pKH32 (*TPI1-lacZ*)		
		Specific β-galact. activity (SD)			Specific β-galact. activity (SD)		
		R (glu)	D (lac)	I (gal)	R (glu)	D (lac)	I (gal)
JS167	Wild-type	6 (1)	104 (22)	2440 (325)	5240 (770)	3125 (445)	4380 (480)
JS05.2–8	*cyc8*∆	3 (< 1)	15 (4)	139 (32)	3870 (865)	2360 (790)	2720 (590)
JS95.7–1	*tup1*∆	15 (5)	327 (77)	1205 (280)	4585 (1065)	3450 (880)	4425 (790)

*SD* standard deviation

Isogenic strains were transformed with reporter plasmids pKH29 (*GAL1–lacZ*) or pKH32 (*TPI1-lacZ*) and subsequently cultivated under repressing (R; 2% glucose, glu), derepressing/non-inducing (D; 0.1% glucose + 1% lactate, lac), and inducing conditions (I; 2% galactose, gal) until mid-exponential growth phase (about 10^7^ cells/ml). In the non-inducing medium, glucose (0.1%) became completely consumed before cell harvest. Specific β-galactosidase activities are given in nmol oNPG hydrolyzed per min per mg of protein

## Discussion

Although there is no complete agreement on all regulatory aspects of *GAL* gene control in *S. cerevisiae*, Gal80 is generally considered as an antagonist of Gal4, needed to shield its activation domain unless galactose induction becomes effective.

**Fig. 5:**
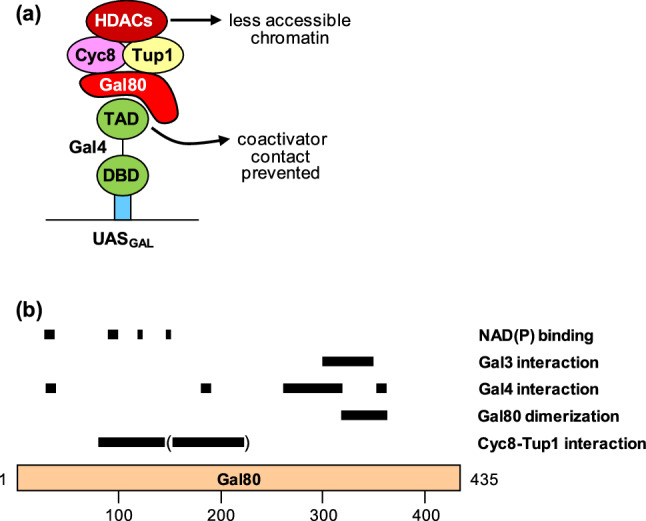
**a** Modified hypothesis of *GAL* gene control postulating a dual mechanism of repression under non-inducing (derepressing) growth conditions. For simplicity of the model, the dimeric structures of Gal4 and Gal80 and the correct stoichiometry of the Cyc8–Tup1 complex are not shown. **b** Summary of protein–protein and protein–nucleotide interactions described for Gal80. Conformation of Gal80 was found to be tripartite, consisting of a N-terminal Rossmann-fold with a helix/sheet structure required for binding of NAD(P), a C-terminus of nine β-sheets important for dimer formation and a central cleft separating these domains which is involved in binding of the Gal4 activation domain. Structural and mutational data were taken from Yano and Fukasawa (1997), Melcher (2005), Thoden et al. (2007, 2008), Kumar et al. (2008), and Lavy et al. (2012)

Here, we provide evidence that this is not the sole function of Gal80 but that a second mechanism may prevent maximal expression of UAS_GAL_-containing genes under non-inducing (derepressing) conditions. We show that Gal80 is able to interact with corepressor proteins Cyc8 and Tup1 which are known to recruit several histone deacetylases (Rpd3, Hda1, Hos1, and Hos2; Davie et al. 2003; Watson et al. 2000; Wu et al. 2001). Gal80 not only contacts Cyc8 and Tup1 physically but could also efficiently repress gene expression in vivo when a *lexA–GAL80* fusion gene was transformed into a strain with a reporter gene containing lexA operator sequences in its upstream region. Such a result would not be expected if Gal80 would simply prevent interaction of Gal4 with pleiotropic coactivators. Our results instead provide evidence that a more active mechanism of Gal80-dependent repression independent of Gal4 is also effective in the control of the *GAL* regulon. We thus suggest that interaction of Gal80 with Cyc8–Tup1 allows associated HDACs to hamper access of general transcription factors to local chromatin. The dual mode of repression proposed here is depicted in Fig. 5a, summarizing regulatory interactions under non-inducing conditions. As is evident by its genetic and physical interaction with the Srb10 CTD kinase module, Cyc8–Tup1 may also prevent gene expression by inhibition of the mediator complex (Kuchin and Carlson 1998; not investigated in this work).

Gal80 has been intensively characterized by identification of mutations which abolish interaction with Gal4 (repression-defective; Melcher 2005) or with Gal3 (non-inducible; Yano and Fukasawa 1997) as well as by structural studies (Thoden et al. 2007, 2008; Kumar et al. 2008; Lavy et al. 2012). As a result of these investigations, separate functional Gal80 domains involved in dimerization, interaction with inducer Gal3 and activator Gal4, and binding of nucleotide NAD(P) have been defined (summarized in Fig. 5b). In this work, we were able to identify a new Gal80 domain of 65 amino acids (aa 81-145) which interacted with Cyc8 and Tup1 in vitro and also strongly mediated gene repression in vivo (lexA-Gal80_81-145_). Site-directed mutagenesis of this domain at selected positions emphasized the importance of aromatic-hydrophobic amino acids. Reduced gene repression in vivo was most obvious for mutation of residues Y118, V121, and L125 which form a short hydrophobic-amphipathic sequence. However, we did not identify specific mutant variants which were completely defective for gene repression, indicating that separate positions within aa 81-145 must be involved as well. A neighboring domain (aa 146-220) also contacted Cyc8 and Tup1 in vitro but was substantially less effective in gene repression (shown in parenthesis in Fig. 5b).

Initially, Cyc8 and Tup1 were merely considered to mediate glucose repression of *GAL* genes, being recruited to promoters by interaction with the zinc-finger repressor Mig1 (Treitel and Carlson 1995) which is excluded from the nucleus by the exportin Msn5 following phosphorylation by the Snf1 protein kinase when glucose becomes limiting (DeVit and Johnston 1999). However, Papamichos-Chronakis et al. (2002) could demonstrate that Cyc8 and Tup1 are bound to the *GAL1* upstream region even under conditions of galactose induction. Although most Mig1 was localized to the cytoplasm after its Snf1-dependent phosphorylation, Cyc8–Tup1 did still occupy the *GAL1* promoter under either conditions (Papamichos-Chronakis et al. 2004). In this work, we show by chromatin immunoprecipitation (ChIP) that Cyc8–Tup1 is indeed present at the *GAL1* promoter in a wild-type strain under derepressing conditions, while no binding was observed in a *gal80* single and a *gal80 mig1* double-deletion mutant. In contrast, Cyc8–Tup1 recruitment remained unaffected when only Mig1 was absent. The ability of Cyc8–Tup1 to interact with at least two factors of *GAL* gene control (Mig1, Gal80) explains the continuous presence of the corepressor complex upstream of *GAL1* upon the switch from glucose repression to derepression. Since Tup1 is able to bind even activation domains (shown for VP16 and Gcn4; Wong and Struhl 2011), Gal4 may also be a candidate for corepressor recruitment under inducing conditions. Based on a kinetic analysis of ChIP data, these authors further propose that masking of activation domains and hence inhibition of coactivator access is the major mechanism of gene repression by Cyc8–Tup1.

The complexity of functions described for Cyc8 and Tup1 in combination with the diversity of *cyc8* and *tup1* mutant phenotypes may raise doubts whether the term “corepressor” is a really adequate description. Dual regulators Cyc8–Tup1 not only mediate glucose repression of *GAL* genes but are present at UAS_GAL_-containing promoters even under conditions of galactose induction. As a result of their versatile interaction specificities, Cyc8–Tup1 execute a positive function as well and facilitate Cti6- and phosphatidylinositol-3,5-bisphosphate-dependent recruitment of the histone acetyltransferase complex SAGA (Papamichos-Chronakis et al. 2002; Han and Emr 2011). This dual function is also apparent from the expression studies described in this work. Expression of a *GAL1–lacZ* reporter gene increased in a *tup1* null mutant under repressing and derepressing conditions, while galactose induction remained below the level obtained in the wild-type strain. In contrast, loss of Cyc8 led to reduced expression of *GAL1–lacZ* under all conditions assayed, emphasizing its positive function. Results obtained for other target genes also agree with the hypothesis of a transition of Cyc8–Tup1 from a corepressor to a coactivator (retrograde regulation of *CIT2*, Conlan et al. 1999; Sko1-dependent regulation of osmotic stress, Proft and Struhl 2002). Not only repressor proteins but also activators such as Ino2, Pho4, and Hac1 are able to interact with Cyc8 and Sin3, supporting the view that an extended interpretation of the molecular properties of these pleiotropic “corepressors” is needed (Kliewe et al. 2017).

## Supplementary Information

Below is the link to the electronic supplementary material.

Supplementary file1 (DOCX 67 KB)

## Data Availability

Original data are available upon request. Additional information is provided in the Supplementary Material.
