# Supplementary material for: Transcriptional repressor Gal80 recruits corepressor complex Cyc8–Tup1 to structural genes of the Saccharomyces cerevisiae GAL regulon
Source: Curr Genet. 2021 Oct 7;68(1):115–24. doi: 10.1007/s00294-021-01215-x (PMC8801411; doi:10.1007/s00294-021-01215-x)
Supplement: Supplementary file 1 — Supplementary file1 (DOCX 67 KB) [file 294_2021_1215_MOESM1_ESM.docx]

**Supporting Online Table S1:**

**Strains of *Saccharomyces cerevisiae* used by Lettow et al.:**

| **Strain** | **Genotype** |
| --- | --- |
| C13-ABY.S86 | *MAT*α *ura3 leu2 pra1 prb1 prc1 cps1* |
| FKY12 | *MAT*α *ura3 leu2 his3 pra1 prb1 prc1 cps1 CYC8-His_6_-HA_3_::kanMX* |
| JuLY2 | *MAT*α *ura3 leu2 his3 pra1 prb1 prc1 cps1 CYC8-His_6_-HA_3_::kanMX gal80*Δ*::LEU2* |
| JuLY4 | *MAT*α *ura3 leu2 his3 pra1 prb1 prc1 cps1 CYC8-His_6_-HA_3_::kanMX gal80*Δ*::LEU2 mig1*Δ*::URA3* |
| JuLY7 | *MAT*α *ura3 leu2 his3 pra1 prb1 prc1 cps1 CYC8-His_6_-HA_3_::kanMX mig1*Δ*::URA3* |
| JuLY1 | *MAT*α *ura3 leu2 his3 pra1 prb1 prc1 cps1 TUP1-His_6_-HA_3_::kanMX* |
| JuLY3 | *MAT*α *ura3 leu2 his3 pra1 prb1 prc1 cps1 TUP1-His_6_-HA_3_::kanMX gal80*Δ*::LEU2* |
| JuLY5 | *MAT*α *ura3 leu2 his3 pra1 prb1 prc1 cps1 TUP1-His_6_-HA_3_::kanMX gal80*Δ*::LEU2 mig1*Δ*::URA3* |
| JuLY8 | *MAT*α *ura3 leu2 his3 pra1 prb1 prc1 cps1 TUP1-His_6_-HA_3_::kanMX mig1*Δ*::URA3* |
| RTS-lexA | *MAT*α *leu2 trp1 his3 ura3::lexA_Op_-CYC1-lacZ ::URA3* |
| JS167 | *MATa ura3* |
| JS05.2-8 | *MATa ura3 leu2 cyc8*Δ*::LEU2* |
| JS95.7-1 | *MATa ura3 trp1 tup1*Δ*::TRP1* |

**Plasmids constructed and used by Lettow et al.:**

| **Plasmid** | **Genotype** |
| --- | --- |
| pCW117 | 2µm *URA3 MET25*_Pr_-HA_3_-*SIN3* (full length; Wagner et al. 2001) |
| pFK76 | tet_Pr/Op_-*HA_3_-TUP1*_1-714_ |
| pFK77 | tet_Pr/Op_-*HA_3_-CYC8*_1-398_ |
| pJL13 | tac_Pr/Op_-*GST-GAL80*_81-145_ |
| pJL14 | tac_Pr/Op_-*GST-GAL80*_146-220_ |
| pJL17 | 2µm *LEU2 MET25*_PR_*-*HA_3_-*lexA*_DBD_-NLS-*GAL80* |
| pJL21 | 2µm *LEU2 MET25*_PR_*-*HA_3_-*lexA*_DBD_-NLS-*GAL80*_81-145_ |
| pJL22 | 2µm *LEU2 MET25*_PR_*-*HA_3_-*lexA*_DBD_-NLS-*GAL80*_146-220_ |
| pJL25 | 2µm *LEU2 MET25*_PR_*-*HA_3_-*lexA*_DBD_-NLS-*GAL80*_90-145_ |
| pJL29 | tac_Pr/Op_-*GST-GAL80*_90-145_ |
| pJL34 | tet_Pr/Op_-HA_3_-*SIN3*_1-480_ |
| pJL35 | 2µm *LEU2 MET25*_PR_*-*HA_3_-*lexA*_DBD_-NLS-*GAL80*_1-145_ |
| pJL36 | 2µm *LEU2 MET25*_PR_*-*HA_3_-*lexA*_DBD_-NLS-*GAL80*_146-290_ |
| pJL37 | 2µm *LEU2 MET25*_PR_*-*HA_3_-*lexA*_DBD_-NLS-*GAL80*_81-145_ F120A |
| pJL38 | 2µm *LEU2 MET25*_PR_*-*HA_3_-*lexA*_DBD_-NLS-*GAL80*_81-145_ W123A |
| pJL40 | 2µm *LEU2 MET25*_PR_*-*HA_3_-*lexA*_DBD_-NLS-*GAL80*_291-436_ |
| pJL42 | 2µm *LEU2 MET25*_PR_*-*HA_3_-*lexA*_DBD_-NLS-*GAL80*_81-145_ K117A |
| pJL43 | 2µm *LEU2 MET25*_PR_*-*HA_3_-*lexA*_DBD_-NLS-*GAL80*_81-145_ Y118A |
| pJL44 | 2µm *LEU2 MET25*_PR_*-*HA_3_-*lexA*_DBD_-NLS-*GAL80*_81-145_ L119A |
| pJL45 | 2µm *LEU2 MET25*_PR_*-*HA_3_-*lexA*_DBD_-NLS-*GAL80*_81-145_ V121A |
| pJL47 | 2µm *LEU2 MET25*_PR_*-*HA_3_-*lexA*_DBD_-NLS-*GAL80*_81-145_ L125A |
| pJL48 | tac_Pr/Op_-*GST-GAL80*_146-290_ |
| pJL52 | tac_Pr/Op_-*GST-GAL80*_291-436_ |
| pJL53 | 2µm *LEU2 MET25*_PR_*-*HA_3_-*lexA*_DBD_-NLS-*GAL80*_81-145_ Y118A L119A F120A |
| pJL55 | tac_Pr/Op_-*GST-GAL80*_81-145_ F120A |
| pJL56 | tac_Pr/Op_-*GST-GAL80*_81-145_ W123A |
| pJL57 | tac_Pr/Op_-*GST-GAL80*_81-145_ K117A |
| pJL58 | tac_Pr/Op_-*GST-GAL80*_81-145_ Y118A |
| pJL59 | tac_Pr/Op_-*GST-GAL80*_81-145_ L119A |
| pJL60 | tac_Pr/Op_-*GST-GAL80*_81-145_ V121A |
| pJL61 | tac_Pr/Op_-*GST-GAL80*_81-145_ L125A |
| pJL62 | tac_Pr/Op_-*GST-GAL80*_81-145_ Y118A L119A F120A |
| pJL63 | tac_Pr/Op_-*GST-GAL80*_81-145_ L119A F120A V121A |
| pJL65 | 2µm *LEU2 MET25*_PR_*-*HA_3_-*lexA*_DBD_-NLS-*GAL80*_81-145_ L119A F120A V121A |
| pJL78 | *gal80*∆*::LEU2* |
| pJN41 | *mig1*∆*::URA3* |
| pKH29 | 2µm *URA3 GAL1-lacZ* (-631 / +3 of *GAL1*) |
| pKH32 | 2µm *URA3 TPI1-lacZ* (-600 / +3 of *TPI1*) |
| pRAR41 | tac_Pr/Op_-*GST-GAL80* |
| pRAR53 | tac_Pr/Op_-*GST-GAL80*_1-145_ |
| pRAR68 | tac_Pr/Op_-*GST-GAL80*_1-100_ |
| pRAR70 | tac_Pr/Op_-*GST-GAL80*_202-290_ |
| pRT-lexA | 2µm *LEU2 MET25*_PR_*-*HA_3_-*lexA*_DBD_-NLS |
| pU6H-3HA | His_6_-HA_3_ *kanMX* |

Pr, promoter; Op, operator.

**Oligonucleotides used by Lettow et al. (PCR primers for strain modifications at locus *TUP1*; quan­tification of ChIP analyses; construction of length variants of gene *GAL80*; site-directed mutagenesis of *GAL80*):**

| **Name** | **Gene** | **Position** | **Sequence 5‘-3‘** |
| --- | --- | --- | --- |
| Act1-FOR3 | *ACT1* | +841/+860 | ATCGATTTGGCCGGTAGAGA |
| Act1-REV3 | *ACT1* | +1156/  +1142 | CACACTTCATGATGGAGTTGTAAG |
| Gal1-F170 | *GAL1* | -509/-489 | CCTTCTCTTTGGAACTTTCAG |
| Gal1-R450 | *GAL1* | -214/-233 | ATCGCATTATCATCCTATGG |
| Gal80 3R aa145 *Hind*III | *GAL80* | +435/+416 | gatc**aagctt**TCATTGAACCCCACGTTCAGCAG |
| Gal80 3R aa201 *Hind*lll | *GAL80* | +603/+584 | gatc**aagctt**TCACCCGATTTCATAGATGTATT |
| Gal80 3R aa220 *Hind*III | *GAL80* | +660/+639 | gatc**aagctt**TCAGTATTGTAAAATATCGATTGTG |
| Gal80 3R aa290 *Hind*III | *GAL80* | +870/+854 | gatc**aagctt**TCATTTGGTAAATTTTTTGG |
| Gal80 3R aa436 *Hind*III | *GAL80* | +1308/  +1289 | gatc**aagctt**TCATTATAAACTATAATGCGAGA |
| Gal80 5F aa1 *Bam*HI | *GAL80* | +1/+20 | gatc**ggatcc**ATGGACTACAACAAGAGATC |
| Gal80 5F aa146 *Bam*Hl | *GAL80* | +436/455 | gatc**ggatcc**ACCATCATCTCTTTACAAGG |
| Gal80 5F aa291 *Bam*HI | *GAL80* | +871/+852 | gatc**ggatcc**AATTTGGTCATTGACATTCA |
| Gal80 5F aa81 *Bam*HI | *GAL80* | +241/+261 | gatc**ggatcc**TTTGCATCATCTTCCACTATA |
| Gal80 5F aa90 *Bam*HI | *GAL80* | +268/+280 | gatc**ggatcc**ATAGTGATAGCTATCCAAGTGGC |
| Gal80 aa81-145 5F K117A | *GAL80* | +328/+372 | TCCAAAAATAATCCGAACCTCGCGTATCTTTTCGTAGAATGGGCC |
| Gal80 aa81-145 3R K117A | *GAL80* | +372/+328 | GGCCCATTCTACGAAAAGATACGCGAGGTTCGGATTATTTTTGGA |
| Gal80 aa81-145 5F Y118A | *GAL80* | +331/+375 | AAAAATAATCCGAACCTCAAGGCTCTTTTCGTAGAATGGGCCCTT |
| Gal80 aa81-145 3R Y118A | *GAL80* | +375/+331 | AAGGGCCCATTCTACGAAAAGAGCCTTGAGGTTCGGATTATTTTT |
| Gal80 aa81-145 5F L119A | *GAL80* | +334/+378 | AATAATCCGAACCTCAAGTATGCTTTCGTAGAATGGGCCCTTGCA |
| Gal80 aa81-145 3R L119A | *GAL80* | +378/+334 | TGCAAGGGCCCATTCTACGAAAGCATACTTGAGGTTCGGATTATT |
| Gal80 aa81-145 5F F120A | *GAL80* | +335/+384 | ATAATCCGAACCTCAAGTATCTTGCCGTAGAATGGGCCCTTGCATGTTCA |
| Gal80 aa81-145 3R F120A | *GAL80* | +384/+335 | TGAACATGCAAGGGCCCATTCTACGGCAAGATACTTGAGGTTCGGATTAT |
| Gal80 aa81-145 5F V121A | *GAL80* | +340/+384 | CCGAACCTCAAGTATCTTTTCGCAGAATGGGCCCTTGCATGTTCA |
| Gal80 aa81-145 3R V121A | *GAL80* | +384/+340 | TGAACATGCAAGGGCCCATTCTGCGAAAAGATACTTGAGGTTCGG |
| Gal80 aa81-145 5F W123A | *GAL80* | +345/+394 | CCTCAAGTATCTTTTCGTAGAAGCGGCCCTTGCATCTTCACTAGATCAAG |
| Gal80 aa81-145 3R W123A | *GAL80* | +394/+345 | CTTGATCTAGTGAACATGCAAGGGCCGCTTCTACGAAAACATACTTGAGC |
| Gal80 aa81-145 5F L125A | *GAL80* | +352/+396 | TATCTTTTCGTAGAATGGGCCGCTGCATGTTCACTAGATCAAGCC |
| Gal80 aa81-145 3R L125A | *GAL80* | +396/+352 | GGCTTGATCTAGTGAACATGCAGCGGCCCATTCTACGAAAAGATA |
| Gal80 aa81-145 5F  Y118A L119A F120A | *GAL80* | +331/+381 | AAAAATAATCCGAACCTCAAGGCTGCTGCTGTAGAATGGGCCCTTGCATGT |
| Gal80 aa81-145 3R  Y118A L119A F120A | *GAL80* | +381/+331 | ACATGCAAGGGCCCATTCTACAGCAGCAGCCTTGAGGTTCGGATTATTTTT |
| Gal80 aa81-145 5F  L119A F120A V121A | *GAL80* | +334/+384 | AATAATCCGAACCTCAAGTATGCTGCTGCTGAATGGGCCCTTGCATGTTCA |
| Gal80 aa81-145 3R  L119A F120A V121A | *GAL80* | +384/+334 | TGAACATGCAAGGGCCCATTCAGCAGCAGCATACTTGAGGTTCGGATTATT |
| Gal80 ORF | *GAL80* | +991/+1010 | CTAGCCAATGGACAACAAGC |
| Gal80 VER | *GAL80* | -120/-101 | CATGGTTGAGCAAACCTATC |
| Tup1-TagF | *TUP1* |  | GCGGTGATTGTAAAGCAAGGATTTGGAAGTATAAAAAATAGCGCCAAATTCCCACCACC |
| Tup1-TagR | *TUP1* |  | CACAGGAAAAGGAGGGGAAGGGATGAATGGTGAGGAAAGTAACTGTTTGTACTATAGGGA |
| Tup1-Tag-Ver | *TUP1* |  | CAAGGACTCTGTTTATAGCG |

Artificially inserted cleavage sequences for restriction enzymes are shown in **bold**; capital letters represent genuine gene-specific sequences; capital letters underlined indicate sequences introducing site-specific mutations.
